# Monitoring SARS-CoV-2 Seroprevalence in Domestics and Exotic Animals in Southern France

**DOI:** 10.3390/tropicalmed8090426

**Published:** 2023-08-25

**Authors:** Bachirou Tinto, Justine Revel, Laurie Virolle, Baptiste Chenet, Florence Reboul Salze, Alix Ortega, Marielle Beltrame, Yannick Simonin

**Affiliations:** 1 Centre MURAZ, Institut National de Santé Publique (INSP), Bobo-Dioulasso 01, Burkina Faso; tintobachirou@yahoo.fr; 2 Pathogenesis and Control of Chronic Infections, University of Montpellier, INSERM, Etablissement Français du Sang, 34394 Montpellier, France; justine.revel@etu.umontpellier.fr; 3 Parc de Lunaret—Zoo de Montpellier, 34090 Montpellier, France; laurie.virolle@ville-montpellier.fr (L.V.); baptiste.chenet@ville-montpellier.fr (B.C.); 4 Veterinary Clinic des Étangs, 34470 Perols, France; labatut.florence@gmail.com; 5 Sigean African Reserve, 11130 Sigean, France; zoologique@reserveafricainesigean.fr (A.O.);

**Keywords:** SARS-CoV-2, dogs, cats, exotic animals, seroprevalence

## Abstract

Since late 2019, Severe Acute Respiratory Syndrome Coronavirus 2 (SARS-CoV-2) has emerged as a significant global threat to public health. Responsible for the COVID-19 pandemic, this new coronavirus has prompted extensive scientific research to comprehend its transmission dynamics, especially among humans. However, as our understanding deepens, it becomes increasingly clear that SARS-CoV-2’s impact goes beyond human populations. Recent investigations have illuminated the transmission of the virus between humans and various animal species, raising important questions about zoonotic spillover events and their potential implications for both human and animal health. Our study set out to investigate the prevalence of SARS-CoV-2 in domestic animals (dogs and cats) and zoo animals in the south of France in 2021 and 2022, covering pre-Omicron and Omicron waves. We identified evidence of SARS-CoV-2 antibodies not only in domestic dogs and cats but also in several mammals in zoos. This study shows the importance of implementing surveillance measures, including serological studies, to identify and monitor cases of SARS-CoV-2 infection in animals.

## 1. Introduction

Severe Acute Respiratory Syndrome Coronavirus 2 (SARS-CoV-2) is a member of the Coronaviridae family, belonging to the genus Betacoronavirus. It is the causative agent of the coronavirus disease 2019 (COVID-19) pandemic, a global health crisis that has profoundly affected human societies and healthcare systems. The pandemic of SARS-CoV-2 began in late 2019 in the city of Wuhan, Hubei Province, China, when an alarming cluster of pneumonia cases with an unknown cause was reported [1]. The pathogen was swiftly identified as a novel coronavirus, genetically related to the viruses responsible for the severe acute respiratory syndrome (SARS) outbreak in 2002–2003 and the Middle East respiratory syndrome (MERS) outbreak in 2012, both of which originated from animal reservoirs before spilling over into human populations [2,3]. However, unlike its predecessors, SARS-CoV-2 displayed distinct genetic characteristics and epidemiological features, enabling it to rapidly disseminate across international borders. SARS-CoV-2 can spread through three primary routes: direct contact with infected secretions (such as saliva and respiratory secretions), droplet transmission (occurring during coughing or sneezing), and aerosol transmission [4]. The initial outbreak in a seafood market in Wuhan, China raised concerns about zoonotic transmission, as many early cases were linked to individuals with exposure to live animals. As investigations progressed, scientists identified bats as a potential natural reservoir for SARS-CoV-2, with the virus possibly jumping to humans via an as yet unidentified intermediate animal host [5]. This zoonotic origin highlights the interconnectedness between humans and wildlife and underscores the importance of understanding the dynamics of transmission between these populations. While humans remain the primary reservoir for sustained transmission, the virus has demonstrated an ability to cross the species barrier. The mechanisms of transmission from humans to animals are still being investigated. Close contact with infected individuals, such as household exposure, appears to be the primary route of transmission. Additionally, contaminated environments and fomites may play a role in spreading the virus between species.

The transmission of SARS-CoV-2 to animals has been reported in various settings, including domestic and wildlife animals [6,7,8,9,10,11,12,13,14,15,16,17,18,19,20,21,22,23]. Instances of infections in companion animals such as dogs, cats, and ferrets and in livestock animals such as cows and cattle have been documented, raising concerns about potential reverse zoonosis—spillover events from infected humans to animals [6,7,8,9,10,11,12,13,14,15,16,17,18,19,20,21,22,23]. Pet infections are likely underestimated, primarily because most infected animals do not display clinical signs. Experimental infections have indicated that the majority of companion animals experience transient infections as evidenced by PCR positivity or virus isolation, hence the importance of seroprevalence studies with a broader detection period [24,25].

Zoonotic SARS-CoV-2 transmission is not associated with domestic animals only, and the transmission events include both captive and wild animals [3,26,27]. Moreover, outbreaks on mink farms have highlighted the potential for virus transmission among densely housed animals, prompting serious public health and animal welfare concerns [28]. It remains crucial to differentiate between incidental infections originating from human-to-animal transmission and sustained animal-to-animal transmission. Understanding the transmission dynamics of SARS-CoV-2 to animals is critical for several reasons. First, it provides insights into the potential reservoirs and intermediate hosts involved in the virus spillover events. Second, it helps assess the risk of viral persistence and potential re-introduction into the human population from animal sources. Additionally, the health and welfare implications for animals infected with SARS-CoV-2 require careful consideration.

Given the global occurrence of infected pets and animals in zoos, it is important to monitor the presence of SARS-CoV-2 in animals. Additionally, the considerable diversity of zoo animals, both in terms of taxonomy and geographical origin, makes zoos an ideal setting to contribute to understanding the potential host range of SARS-CoV-2 and to assess the risk it poses to the conservation of wild animal populations, both in captivity and in their natural habitats. For this study, we conducted serological investigations on blood samples from cats, dogs, and exotic animals from two zoos in the Occitanie region (southern France) in 2021 and 2022, covering several waves of human infections involving different SARS-CoV-2 variants.

## 2. Materials and Methods

### 2.1. Sample Collection

#### 2.1.1. Domestic Animals

Serum samples were collected from 384 pets (mainly indoor pets, 201 dogs and 184 cats) in 2019 (pre-pandemic), 356 dogs and 243 cats in 2021, and 315 dogs and 212 cats in 2022. Samples were collected by convenience sampling, with no mathematical formulas used for their calculation. They came from animals undergoing health evaluations or surgical interventions at veterinary clinics that serve animals from across the Montpellier area (Southern France) without setting symptom criteria. Serum samples were stored at −20 °C until being processed.

#### 2.1.2. Exotic Animals

Montpellier zoo covers an area of 80 hectares and is home to some 1000 animals representing 106 species, including birds and mammals. The Sigean African Reserve is home to more than 3800 animals of 160 different species over more than 300 hectares. The sera were collected at these zoos between 2016 and 2022. In total, 417 sera belonging to 44 orders of mammals were analyzed. For each sample, a volume ranging from 0.3 to 3 mL of blood was obtained from either the cutaneous ulnar vein, the jugular vein, or a femoral vein. The collected blood was then centrifuged at 2000× *g* for 10 min to separate the serum from the clot. The serum was subsequently stored at −20 °C until it was ready for analysis.

#### 2.1.3. Ethical Statement

All experimental procedures were conducted in accordance with national regulations. Veterinary professionals from the zoos and veterinary clinic performed all the sample collection. The samples were sourced from existing serum banks or obtained during routine health check-ups, sanitation programs, or surgical interventions on the animals. No animals were specifically sampled solely for the purpose of this study.

### 2.2. Indirect ELISA Test for the Detection of SARS-CoV-2 Antibodies

The serological status of the animals was evaluated using a commercially available SARS-CoV-2 N double antigen enzyme-linked immunosorbent assay (ELISA) kit following the manufacturer’s instructions (ID Screen^®®^ SARS-CoV-2 Double Antigen Multi-species ELISA, ID.vet, Grabels, France). This ELISA specifically detects IgG antibodies against the nucleocapsid (N) protein of the SARS-CoV-2 virus in animal sera and was conducted as per the manufacturer’s recommended protocol. In summary, 25 µL of each serum sample was diluted at a 1:1 ratio with the dilution buffer and added to each well of a 96-well plate. The plate was then incubated at 37 °C for 45 min. Following incubation, the plate was washed with 300 µL of washing solution, and 100 µL of N protein recombinant antigen horseradish peroxidase (HRP) conjugate was added to each well and incubated at 25 °C for 30 min. After another round of washing (3 times with 300 µL of washing solution), 100 µL of the substrate solution was added to each well and incubated at 25 °C for 20 min. Finally, 100 µL of the stop solution was added to terminate the reaction, and the optical densities (OD) at 450 nm were read and recorded. The OD value of each sample was then calculated as the S/P percentage (S/P%). A sample with S/P% ≥ 60% was considered positive for SARS-CoV-2 antibodies, while a sample with S/P% between 50% and 60% was considered suspected, and a sample with S/P% < 50% was deemed negative.

### 2.3. Surrogate Virus Neutralization Test for the Detection of SARS-CoV-2 Antibodies

The cPass SARS-CoV-2 Neutralization Antibody Detection Kit (GenScript Biotech, Nanjing, China) was employed to identify neutralizing antibodies against SARS-CoV-2. This assay works by measuring the inhibition of the SARS-CoV-2 RBD-ACE2 interaction mediated by antibodies. To outline the procedure briefly, 50 µL of serum diluted at a 1:10 ratio was incubated with 50 µL of HRP-conjugated RBD at 37 °C for 30 min. Next, the treated serum (100 µL) was added to an ACE2-coated ELISA plate and further incubated at 37 °C for 15 min. Subsequently, the uncaptured substrate was removed by washing with 260 µL of washing solution four times. The colorimetric signal was generated using TMB substrate at 25 °C for 15 min. An absorbance reading at 450 nm was taken using a microplate reader immediately after adding the stop solution. The percentage inhibition was then calculated, and samples with a percentage inhibition ≥20% were considered positive for the presence of SARS-CoV-2-neutralizing antibodies, while those with a lower percentage were deemed negative [29].

### 2.4. RT-qPCR

Viral RNAs were extracted from 50 μL of serum with the EZ1 apparatus running the EZ1 DSP virus kit (Qiagen, (Hilden, Germany)). Viral RNA levels were measured by quantitative reverse transcriptase PCR assay (RT-qPCR) on a LightCycler 480 real-time PCR apparatus (Roche) using ID™ SARS-CoV-2 Fast Essential Triplex, a ready-to-use kit that allows SARS-CoV-2 detection (ID Solutions, Grabels, France) [30,31].

### 2.5. Statistical Analysis

The seroprevalence of SARS-CoV-2 was determined by calculating the ratio of positive animals to the total number of animals tested, along with two-sided exact binomial 95% confidence intervals (95% CI) to estimate the precision of the prevalence estimate. To investigate potential correlations between seroprevalence and other independent variables such as sex, age, etc., we employed either a Pearson’s chi-square test or the Fisher’s exact test when the sample size in a group was less than 6.

## 3. Results

### 3.1. SARS-CoV-2 Seroprevalence in Dogs and Cats

The survey was conducted from January 2021 to October 2022. During this period, France was confronted with several waves of SARS-CoV-2, mainly involving the alpha variant in early 2021, which was replaced by the delta variant in mid-2021. The much more transmissible Omicron variant appeared at the end of November 2021 and became the main variant in France in January 2022. We first analyzed the seroprevalence of SARS-CoV-2 by ELISA in 599 pets sampled in 2021, including 356 dogs and 243 cats. Among these samples, nine dogs were tested positive for SARS-CoV-2 antibodies (2.5%; 95% CI: 0.9–4.2) and eight cats (3.3%; 95% CI: 1.0–5.5), representing 2.8% of positives for all pets (CI: 1.5–4.2) (Table 1). In 2022 (mainly Omicron variants), 527 pets were analyzed including 315 dogs and 212 cats. We identified antibodies against SARS-CoV-2 in 32 of the 527 samples (6.1%; 95% CI: 4.0–8.1), representing 15 positives for dogs and 17 for cats (4.7%; 95% CI: 2.4–7.1; 8%; 95% CI: 4.3–11.4). The same analysis conducted on 384 pets over the pre-COVID period (2019) did not reveal any positive samples by ELISA. In addition, all ELISA-positive samples were screened by the Virus Neutralization Test (sVNT) using the cPass™ SARS-CoV-2 Neutralization Antibody Detection Kit, giving positive results for 38 of the 44 samples analyzed. No statistically significant association was observed between seropositivity and the sex or breed of the dogs and cats. Blood samples from pets showing positive seroprevalence were additionally analyzed by RT-PCR for the presence of SARS-CoV-2 RNA, but we did not identify an acute viral infection in these available samples. We found that the seroprevalence of SARS-CoV-2 appears to be higher in cats than in dogs, although the number of positive samples is too low to deduce a statistically significant trend. Moreover, the study shows a higher number of positive samples in 2022 compared with 2021 (6.1 versus 2.8), in both dogs and cats, probably due to the circulation of the Omicron variant in 2022 (including mainly in the BA.1 sublineage progressively replaced at the middle of the year by BA.4 and BA.5 sublineages). This variant is known to be more contagious and is now the main variant worldwide. The majority of the seropositive animals studied had no symptoms suggestive of SARS-CoV-2 infection. We did, however, identify a 9-year-old cat with fatigue and coughs and a 13-year-old boxer dog with the same symptoms, but for these animals, the etiology could not be determined at the time of consultation.

### 3.2. SARS-CoV-2 Seroprevalence in Exotic Mammals

It should be noted that no mortality related to viral infection occurred at Montpellier Zoo during the analysis period. All 212 sera collected pre-pandemic (2016–2018) were seronegative by ELISA. The study of seroprevalence in mammals during the pandemic (2021–2022) was performed on 205 specimens of 44 different species (Table 2). A total of 8 mammals were found positive by the ELISA kit (3.9%, CI 95%: 1.3–6.6), including three springbok (*Antidorcas marsupialis*) and three Cameroon sheep (*Ovis aries cameroon*), including two confirmed by sVNT and two Vicuna (*Vicugna vicugna*) confirmed by sVNT. Thus, according to the available data, only a small proportion of exotic mammal species was exposed to SARS-CoV-2 infections, probably due to limited contact with humans. Susceptibility to infection has already been documented in sheep and small ruminants. Our study is the first to identify springbok and vicuna as susceptible to SARS-CoV-2 infection [32,33]. The first belonging to the bovidae family and the second to the camelidae family. No statistically significant associations between seropositivity and sex or age were observed. As for pets, we did not identify acute viral infection in the seropositive samples analyzed by RT-PCR for the presence of SARS-CoV-2 RNA.

## 4. Discussion

During the collection period of our samples, France was confronted with a wave of the alpha variant at the beginning of 2021, then a wave of the delta variant in mid-2021. The most easily transmissible Omicron variant caused three major waves in 2022 over the study period, involving different Omicron subligneages. The epidemiological situation in our study area closely mirrored that of the rest of the country during the initial waves. As the pandemic advanced and human cases surged globally, there was a notable rise in seroprevalence among companion animals. A study established a correlation between the density of SARS-CoV-2 infections in humans and the number of seropositive animals, indicating a connection as the infection spread between species [20].

While most animal infections are of human origin, the specific risk factors for zoonotic transmission from humans to animals and the frequency and characteristics of clinical illness in animals are not yet well defined. Since the initial report of a SARS-CoV-2 infection in a dog in March 2020 [34], an expanding array of species has been found to be vulnerable to infection. Mounting evidence indicates the significance of animals in SARS-CoV-2 transmission. The introduction of the virus into mink farms led to local epidemics in these highly vulnerable animals, with reports of mink-to-human spillback infections raising concerns about viral persistence in animals [28]. This is also the case for white-tailed deer, where frequent intraspecies transmission has been documented, posing the possibility of establishing animal reservoirs that could perpetually pose a risk for back-transmission of the virus to humans [35,36,37]. As of now, SARS-CoV-2 spillover from humans to animals has been documented in more than 30 countries across at least 17 animal species, including pets like cats, dogs, ferrets, and hasmters, but also livestock and exotic species like otters, lions, tigers, pumas, snow leopards, gorillas, fishing cats, binturongs, coatis, hyenas, lynx, otters, and hippos [3,14,15,26,38,39].

There is a specific risk of exposure to SARS-CoV-2 for domestic dogs and cats due to their close contact with humans, which creates many opportunities for viral exposure. In these pets, SARS-CoV-2 infection has been reported in several countries in America, Europe, including France, and Asia [6,7,8,9,10,11,12,13,16,17,18,19,20,21,22,23]. In a broader context, these studies demonstrate that the transmission of SARS-CoV-2 from humans to companion animals, while not occurring systematically, is not an uncommon phenomenon. However, there are still many unknowns, for example, concerning their susceptibility to the different variants. The emergence of SARS-CoV-2 variants of concern raises an important question about whether the evolution of the virus will lead to an increased likelihood of reverse zoonoses and animal transmission. It is therefore important when new variants of the virus appear to determine the degree of susceptibility of companion animals. Moreover, seroprevalence studies conducted in Europe have shown varying positivity rates [8,9,10,11,19,20,22,23,40,41,42,43,44,45,46,47]. The variations in rates could be attributed to numerous factors, including the number of reported human cases in the area, the period of study, the specific variant in question, the geographical region, and the methodologies employed to encompass animals and ascertain seroprevalence. Infections in animals are generally asymptomatic or may be linked to temporary respiratory or gastrointestinal issues [48,49]. Although death due to SARS-CoV-2 infection has been reported in rare instances, determining its precise contribution in animals with pre-existing conditions like cancer or obesity poses challenges.

Here, we serologically investigated cats and dogs in 2021 and 2022. Our findings indicate that transmission of SARS-CoV-2 from infected humans to their pets, as indicated by seroconversion, is not a rare event. We found 2.8% of positive companion animals in 2021 and 6.1% in 2022. This increase in positivity among pets in 2022 may be associated with the significant increase in the circulation of SARS-CoV-2 in France linked to three successive waves of Omicron variants in the human population. Moreover, we observed a slightly higher seropositivity for cats than for dogs both in 2021 (3.3% versus 2.6%) and in 2022 (8% versus 4.7%). These results align with earlier observations indicating a greater prevalence of SARS-CoV-2 in cats as opposed to dogs [25,50,51]. The relatively elevated susceptibility of cats to SARS-CoV-2 may be attributed to the significant homology between the feline host cell receptor protein angiotensin-converting enzyme 2 (ACE2) and its human counterpart. This homology results in a moderate to strong affinity for the S protein of SARS-CoV-2 in cats, while for dogs, it has been discussed that reduced susceptibility to SARS-CoV-2 could be due to low expression of ACE2 in the airways of dogs [52,53]. Moreover, experimental studies have demonstrated that cats are susceptible to SARS-CoV-2 infection. When exposed to the virus, cats can become infected and have the ability to transmit the disease through respiratory droplets and direct contact with other felines [50]. Risk factor analyses identified plausible associations presumably linked to the duration and closeness of human–animal contact, and there is evidence to suggest that specific human–animal interactions, such as kissing the pet or having the pet sleep on the bed, may increase the risk of transmission [54]. It should be noted that this pet infection can potentially, in very rare cases, lead to a human infection [55]. Infected pets do not necessarily show clinical signs; several reports describe subclinically infected cats with no evidence of clinical signs. Only a small number of case studies documenting natural infection in cats and dogs have shown severe clinical outcomes. These studies indicate that co-morbidities likely played a contributing role in the illness or death observed in these animals [12,18]. The predominant clinical manifestations of SARS-CoV-2 infection in cats are generally very mild and primarily involve the upper respiratory system. These signs include coughing, discharge from the nose and eyes, sneezing, slight lethargy, reduced appetite, and digestive issues such as anorexia and vomiting [7,17,56].

In our study, across all animals that were positive for SARS-CoV-2 antibodies, we identified two pets suffering from fatigue and cough whose initial causes could not be determined. However, the association of the symptoms with SARS-CoV-2 infection could not be determined due to PCR negativity and the absence of nasal swabs to determine the presence of the virus at the time of symptoms. No PCR-positive animals were found in our study, which is not surprising given the design of our study, which is not based on symptom detection. Seroprevalence studies generally give higher values than PCR positivity. This result was expected since serological data reflect past exposure and not acute disease.

Zoonotic transmission of SARS-CoV-2 is not limited to domestic animals exclusively. Some studies have shown that exotic species can also be infected by SARS-CoV-2. Zoos play a crucial role in conservation, research, and education by housing diverse animal species in close proximity to each other and humans, including veterinarians and other zoo workers. They can thus contribute to a better understanding of the species susceptible to SARS-CoV-2 infection. During the early stages of the COVID-19 epidemic in New York City, the initial SARS-CoV-2 outbreak was detected and reported at the Bronx Zoo. This outbreak led to a self-limiting disease affecting three lions and five tigers [57,58]. In contrast to the largely asymptomatic infections observed in domestic animals, these felines exhibited mild to moderate upper respiratory clinical signs. There was a suspicion that these captive wild felids contracted the infection from a zookeeper/worker who tested positive for COVID-19, and cases of SARS-CoV-2 infections in zoo animals were previously linked to asymptomatic zookeepers who had come into contact with these animals [2,58]. The risk of transmission is greatest when zookeepers come into close contact with animals during activities such as food preparation, veterinary consultations, or enclosure cleaning. Based on the open-access dataset of reported SARS-CoV-2 events in animals, approximately 120 zoo animals have been identified with SARS-CoV-2 infections, involving 17 different species [59]. However, the detection of SARS-CoV-2 infections in zoo animals has primarily relied on observing symptoms or instances of death in these captive animals. SARS-CoV-2 infections can go undetected if animals show no obvious symptoms. This reinforces the need for seroprevalence studies.

In our work, a total of eight mammals were found positive by the ELISA kit, including three springboks (*Antidorcas marsupialis*), three Cameroon sheep (*Ovis aries cameroon*), and two Vicuna (*Vicugna vicugna*). None of these species had previously been identified as susceptible to SARS-CoV-2 infection. By performing a comparative analysis of ACE2 protein and genome sequences, it is possible to hypothesize the ability of different species to bind to the SARS-CoV-2 spike protein [60,61]. This approach could provide interesting information on the potential zoonotic transmission of the SARS-CoV-2 infection. These studies determined the risk for Vicuna and Ovis aries as medium, whereas the risk for dogs is deemed to be low, while for cats, it is considered to be high [3,60,61]. Although these studies are useful in assessing the potential host range of SARS-CoV-2, the actual natural infections have revealed that susceptibility solely determined by the ACE2 receptor alone is not sufficient for estimating the potential spillover risk to other species. An illustrative example is the case of wild white-tailed deer, which are not deemed highly susceptible according to these in silico analyses.

In summary, this study provides evidence of SARS-CoV-2 antibodies not only in domestic dogs and cats but also in several mammals in zoos. Comprehending the factors influencing SARS-CoV-2 transmission to animals is of utmost importance, not only for gaining insights into the virus ecology but also for effectively managing the risks it presents to both animal and human populations. Efforts to track and monitor cases of SARS-CoV-2 infection in animals have been put into action including diagnostic testing, genetic sequencing, and epidemiological investigations. These efforts must continue, not only for SARS-CoV-2 but also for other emerging viruses, applying the One Health concept to better understand and anticipate these emergences.

## Figures and Tables

**Table 1:** SARS-CoV-2 seroprevalence in domestic species.

	Dog	Cat	All Pets
2021 Nb of samples	356	243	599
Nb of positive (Elisa)	9	8	17
Total (%)	2.5%	3.3%	2.8%
2022			
Nb of samples	315	212	527
Nb of positive (Elisa)	15	17	32
Total (%)	4.7%	8%	6.1%

**Table 2:** Zoo mammal species tested for exposure to SARS-CoV-2 infection.

Species (English Name)	Species (Scientific Name)	Collection Date	Nb of Specimen	Number of Positive Elisa	sVNT
Cheetah	*Acinonyx jubatus*	2022	1	Neg	/
Addax	*Addax nasomaculatus*	2021	11	Neg	/
Impala	*Aepyceros melampus*	2021	5	Neg	/
Springbok	*Antidorcas marsupialis*	2021	11	3	3
Watusi	*Bos taurus primigenius.*	2022	1	Neg	/
Mindoro dwarf buffalo	*Bubalus mindorensis*	2021	1	Neg	/
Dromedary	*Camelus dromedarius*	2021	2	Neg	/
Iberian wolf	*Canis lupus signatus*	2021	2	Neg	/
White rhinoceros	*Ceratotherium simum*	2021–2022	5	Neg	/
Sika deer	*Cervus nippon*	2021	8	Neg	/
Maned wolf	*Chrysocyon brachyurus*	2021–2022	3	Neg	/
Blue wildebeest	*Connochaetes taurinus*	2022	4	Neg	/
Blesbok	*Damaliscus pygargus phillipsi*	2021	3	Neg	/
Grévy’s zebra	*Equus grevyi*	2021–2022	9	Neg	/
Persian onager	*Equus hemionus onager*	2021	5	Neg	/
Tibetan Wild Ass	*Equus kiang*	2021	1	Neg	/
Grant’s zebra	*Equus quagga boehmi*	2021	7	Neg	/
Hartmann’s mountain zebra	*Equus zebra hartmannae*	2021–2022	7	Neg	/
Cuvier’s gazelle	*Gazella cuvieri*	2022	1	Neg	/
Reticulated giraffe	*Giraffa reticulata*	2022	2	Neg	/
Hippotrague rouan	*Hippotragus equinus*	2022	2	Neg	/
Waterbuck	*Kobus ellipsiprymnus*	2021	3	Neg	/
Southern lechwe	*Kobus leche*	2021	4	Neg	/
Nile lechwe	*Kobus megaceros*	2021–2022	3	Neg	/
African wild dog	*Lycaon pictus*	2021	3	Neg	/
Barbary macaque	*Macaca sylvanus*	2021	1	Neg	/
Bennett’s wallaby	*Macropus rufogriseus*	2022	7	Neg	/
Dama gazelle	*Nanger dama*	2021–2022	8	Neg	/
Scimitar_horned oryx	*Oryx dammah*	2021	1	Neg	/
South African oryx	*Oryx gemsbok*	2021	1	Neg	/
Somali black headed sheep	*Ovis aries*	2021	3	Neg	/
Cameroon Shee	*Ovis aries cameroon*	2021	41	3	2
Corsican Mouflon	*Ovis aries musimon*	2021	3	Neg	/
Atlas lion	*Panthera leo leo*	2022	4	Neg	/
Asian lion	*Panthera leo persica*	2021	2	Neg	/
Warthog	*Phacochoerus aethiopicus:*	2022	2	Neg	/
Bharal	*Pseudois nayaur*	2021	1	Neg	/
Nyala	*Tragelaphus angasii*	2021	3	Neg	/
Eastern bongo	*Tragelaphus eurycerus*	2021	1	Neg	/
Common eland	*Tragelaphus oryx*	2022	5	Neg	/
Tibetan brown bear	*Ursus arctos pruinosus*	2021	1	Neg	/
Syrian brown bear	*Ursus arctos syriacus*	2021	2	Neg	/
Vicuna	*Vicugna vicugna*	2022	5	2	2

## Data Availability

Not applicable.
